# IL-34-mediated fibroblast-like synoviocyte-macrophage crosstalk drives bone erosion in rheumatoid arthritis through RANKL-dependent osteoclastogenesis

**DOI:** 10.3389/fimmu.2026.1822793

**Published:** 2026-06-17

**Authors:** Xin Li, Yurou Yang, Shuao Zhang, Yujing Cai, Aiqi Wang, Na Xu

**Affiliations:** 1 Department of Rheumatology, First Affiliated Hospital of Jinzhou Medical University, Jinzhou, Liaoning, China; 2 Department of Immunization and Prevention, Peng’an County Center for Disease Control and Prevention, Nanchong, Sichuan, China; 3 Department of General Practice, Xinzhuang Town Health Center, Zhaoyuan, Shandong, China; 4 Department of Radiology, The First Affiliated Hospital of Jinzhou Medical University, Jinzhou, Liaoning, China

**Keywords:** fibroblast-like synoviocytes, IL-34, macrophage polarization, osteoclastogenesis, RANKL, rheumatoid arthritis

## Abstract

**Background:**

The crosstalk between fibroblast-like synoviocytes (FLS) and macrophages forms a self-reinforcing vicious feedback loop in the rheumatoid arthritis (RA) immune microenvironment. Upregulated interleukin-34 (IL-34) levels in RA patients correlate with RA severity. This study investigated the roles and mechanisms of IL-34-mediated FLS-macrophage crosstalk in osteoclastogenesis-associated bone erosion in RA.

**Methods:**

IL-34 expression in the RA serum and synovial samples and its correlation with disease severity and bone erosion were assessed. Primary FLS were transfected with IL-34 overexpression plasmids or short hairpin RNA (shRNA) targeting IL-34, and cell proliferation, migration, invasion and apoptosis were examined. Then, conditioned medium (CM) from these FLS were incubated with THP-1-differeniated macrophages to evaluate macrophage migration and polarization. FLS, M1 macrophages, and their cocultures were respectively treated with recombinant human IL-34 (rhIL-34), and these CM were incubated with peripheral blood mononuclear cells (PBMCs). Receptor activator of NF-κB ligand (RANKL) concentration and osteoclast differentiation were determined. Additionally, a collagen-induced arthritis mouse model was established and treated with IL-34 neutralizing antibody. The arthritis score, paw swelling, histopathological analysis micro-CT analysis were conducted.

**Results:**

Upregulated serum IL-34 level in RA patients was correlated with disease severity and bone erosion. IL-34 overexpression facilitated proliferation, migration and invasion and inhibited apoptosis in FLS. Moreover, CM from IL-34-overexpressing FLS promoted macrophage recruitment and M1 polarization. In contrast, IL-34 silencing showed the opposite results. rhIL-34-stimulated FLS-macrophage coculture exhibited higher RANKL production and enhanced osteoclast differentiation. However, RANKL inhibitor denosumab abolished these effects. IL-34 blockade attenuated RA severity, synovial hyperplasia, macrophage M1 polarization, inflammatory infiltration, and bone erosion in CIA mice.

**Conclusion:**

IL-34 accelerates RA progression by regulating FLS-macrophage crosstalk to amplify bone erosion through RANKL-dependent osteoclastogenesis. We suggest that IL-34 blockade as a promising therapeutic strategy for concurrently alleviating synovial hyperplasia, inflammation, and bone erosion in RA.

## 1 Introduction

Rheumatoid arthritis (RA) is a chronic and systemic autoimmune disorder characterized by progressive joint destruction (1, 2). Affecting approximately 0.5-1% of the global population, RA leads to significant disability (3). The onset of RA is closely related to a complex interplay of factors, such as genetics, environment, and immunity, among which dysregulated immune responses are central to RA pathogenesis (4, 5). Its pathogenesis is predominantly defined by synovial hyperplasia, inflammatory infiltration, and articular cartilage destruction, leading to joint deformities and even physical disability (6). The clinical diagnosis of RA hinges on the 2010 ACR/EULAR classification criteria (7) and imaging techniques for the sensitive detection of bone erosion and damage (8). Despite significant advancements in disease-modifying antirheumatic drugs (DMARDs), a notable proportion of patients exhibit limited drug efficacy or drug resistance. Therefore, it is imperative to elucidate the endogenous molecular mechanisms underlying RA pathogenesis and identify valuable therapeutic targets.

Fibroblast-like synoviocytes (FLS) are the primary cellular component of the synovium. Under inflammatory or pathological conditions, FLS becomeabnormally activated and are characterized by hyperproliferation and aggressive migration, which serve as a crucial driver of synovial inflammation and joint destruction in RA progression (9). Hyperproliferative FLS accelerate joint destruction through the excessive secretion of inflammatory cytokines, including tumor necrosis factor alpha (TNF-α), interleukin (IL)-6, and IL-17 (10–12). Furthermore, abnormally activated FLS induce the recruitment and infiltration of immune cells (such as macrophages and T/B lymphocytes) into the synovium by releasing chemokines like C-C motif chemokine ligand 2 (CCL2) and C-X-C motif chemokine ligand 12 (CXCL12) (13, 14). The aberrant immune microenvironment in RA patients disrupts the balance between pro-inflammatory M1 and anti-inflammatory M2 macrophages. Synovial M1 macrophages, a central source of inflammatory cytokines, further enhance FLS activation and osteoclastogenesis (15). Critically, activated FLS recruit and polarize macrophages toward the M1 phenotype, while M1 macrophage-derived inflammatory cytokines further stimulate the aggressive phenotype of FLS, forming a self-reinforcing vicious feedback loop within the RA immune microenvironment (16). This crosstalk between FLS and macrophages drives a self-perpetuating inflammatory cascade marked by the excessive secretion of inflammatory cytokines and matrix metalloproteinases (MMPs), ultimately resulting in articular cartilage degradation. Furthermore, this crosstalk also promotes osteoclastogenesis and amplifies bone erosion by increasing the production of inflammatory cytokines and receptor activator of NF-κB ligand (RANKL) (17). The RANKL-RANK signaling pathway is central to osteoclast differentiation, directly linking synovial inflammation to irreversible bone erosion in RA (18). Therefore, an in-depth exploration of the bidirectional signaling regulatory network between FLS and macrophages is critical for unraveling the molecular mechanisms underlying the vicious inflammatory cycle in RA.

Interleukin-34 (IL-34) is a newly identified pro-inflammatory cytokine, which share a same receptor, colony-stimulating factor 1 receptor (CSF-1R), with macrophage colony-stimulating factor (M-CSF). Consequently, IL-34 exhibits functional similarities to M-CSF in promoting osteoclastogenesis (19). Serum IL-34 levels are upregulated in RA patients, and their expression is positively correlated with disease activity and some standard diagnostic markers (20, 21), serving as a potential biomarker for RA diagnosis. Specifically, serum IL-34 levels are significantly correlated with bone erosion scores and RANKL levels in RA patients (22), suggesting that targeting IL-34 may be a potential therapeutic method for mitigating bone erosion. Furthermore, IL-34 facilitates FLS proliferation and migration and suppresses apoptosis (23, 24), and amplifies pro-inflammatory cytokine production in FLS (25). IL-34 treatment also enhances IL-6 secretion in monocytes/macrophages (26). Notably, TNF-α stimulation increases IL-34 expression in FLS, which further promotes osteoclastogenesis (27). Based on these findings, IL-34 exhibits pleiotropic effects on the activation of FLS, macrophages, and osteoclasts underlying RA pathogenesis.

This study investigates the roles of IL-34-mediated FLS-macrophage crosstalk in inflammatory amplification and osteoclastogenesis in RA, aiming to identify an effective therapeutic target for mitigating irreversible joint damage and provide novel insights into overcoming the limitations of traditional RA therapies.

## 2 Materials and methods

### 2.1 Clinical patients

RA patients (n=42, male = 15, female = 27, average age = 51.9 ± 10.3) were recruited from the First Affiliated Hospital of Jinzhou Medical University. RA was diagnosed according to the American College of Rheumatology criteria (28). Patients undergoing joint surgery due to trauma or non-inflammatory joint diseases (n=35, male = 14, female = 21, average age = 47.8 ± 12.2 years) were recruited as control group during the same period. Patients with other arthritis diseases, malignancy, pregnancy, autoimmune diseases, and other systemic diseases were excluded. Clinical and laboratory data were collected, including disease duration, rheumatoid factor (RF) status, anti−citrullinated protein antibody (ACPA) status, current treatments (conventional synthetic disease−modifying antirheumatic drugs [csDMARDs], biologics, JAK inhibitors, and glucocorticoids), and common comorbidities (hypertension, diabetes). The disease activity was assessed using the Disease Activity Score 28 (DAS28) based on erythrocyte sedimentation rate (DAS28-ESR) and C-reactive protein levels (DAS28-CRP) (29). Radiographic joint damage and bone erosion was assessed with the modified Sharp/van der Heijde (mSvdH) score (30). The baseline demographic and clinical characteristics of both groups are summarized in **Supplementary Table 1**. This study was approved by the Ethics Committee of the First Affiliated Hospital of Jinzhou Medical University, and all patients signed the informed consent. The peripheral blood (approximately 10 mL per patient) and synovial tissue samples (approximately 0.5–1 g per patient) of all participants were harvested from all 42 RA patients and 35 control subjects, and stored in -80°C.

### 2.2 Cell isolation and culture

Primary FLS were isolated through enzymatic dissociation of RA patient-derived synovial tissues. In this study, 6 primary FLS cultures were successfully established from 6 representative RA patient samples for subsequent *in vitro* experiments. Briefly, the synovial tissues were digested in 1% collagenase II with shaking at 37°C for 2 h. Following sequential filtration and centrifugation, the isolated cells were cultured in DMEM containing 10% FBS and 1% antibiotics at 37°C in 5% CO_2_. Passage 3–5 cultures were used for further analyses.

Peripheral blood mononuclear cells (PBMCs) serving as osteoclast precursors for the differentiation assays were isolated from peripheral blood samples of 6 representative healthy controls (n = 6) by density-gradient centrifugation (650 × g, 20 min, 18°C) using Ficoll-Hypaque (Sigma-Aldrich, USA). Approximately 10 mL of peripheral blood was used from each subject, yielding an average of 1.0–1.5 × 10^7^ PBMCs per isolation. PBMCs and THP-1 cells (ATCC) were cultured in complete RPMI1640 medium at 37°C in 5% CO_2_. Medium renewal was conducted at 48-hour intervals. THP-1 cells were differentiated into macrophages by treatment with 100 nM phorbol myristate acetate (PMA) for 24 h, followed by incubation with 100 ng/mL lipopolysaccharide (LPS) and 20 ng/mL interferon-γ (IFN-γ) for 24 h to induce M1 macrophage polarization.

### 2.3 Cell coculture

FLS and M1 macrophage cocultures were prepared were established following a published method (31). To capture biological variability, a total of exactly 6 independent coculture sets (n = 6) were conducted. Specifically, primary FLS derived from 6 individual RA donors were utilized, with each donor representing one independent coculture set. To ensure the reproducibility of THP-1 macrophage polarization without artificially inflating the sample size, the M1 macrophages were prepared in 3 independent differentiation batches. Each independent macrophage batch was paired with FLS from two distinct RA donors (e.g., Macrophage batch 1 was cocultured with FLS from donors 1 and 2; batch 2 with donors 3 and 4; and batch 3 with donors 5 and 6). This strict pairing strategy resulted in exactly 6 distinct coculture sets. Briefly, FLS from each donor were resuspended in DMEM with 10% FBS and seeded in Transwell inserts (3 μm; BD Biosciences, USA) at 2.5×10^4^ cells/cm^2^ for overnight adhesion. Then, M1 macrophages were resuspended in DMEM with 10% FBS and seeded on the FLS layer at 1×10^6^ cells/cm^2^. The recombinant human IL-34 (rhIL-34) (50 and 100 ng/mL) (Catalog #5265-IL, R&D System, USA) was added to the coculture system. Following a 3-day coculture period, the conditioned media (CM) from each of the 6 independent sets was harvested for subsequent experiments. For *in vitro* mechanism studies, PBMCs isolated from 6 individual healthy donors (n = 6) were treated in a one-to-one manner with the CM obtained from the 6 independent 100 ng/mL rhIL-34-stimulated coculture sets (i.e., PBMCs from healthy donor 1 were treated with CM from coculture set 1, PBMCs from donor 2 with CM from set 2, and so forth). To maintain statistical independence, CM samples from each coculture set were used separately to stimulate individual PBMC donors without pooling. Each of the six biological replicates (n = 6) thus represents a unique, independent pairing of an RA-FLS donor and a PBMC donor. This pairing strategy was used to investigate the role of RANKL in IL-34-driven osteoclastogenesis, with or without the addition of the RANKL inhibitor denosumab (10 μg/mL; Sigma-Aldrich, USA).

### 2.4 Lentiviral transduction and stable cell line generation

Lentivirus-carried IL-34 overexpression plasmids (oe-IL-34) and short hairpin RNA (shRNA) targeting IL-34 (sh-IL-34), along with their respective negative controls (empty vector oe-NC and scrambled shRNA sh-NC), were sourced from GenePharma (Shanghai, China). For the construction of oe-IL-34, the full-length coding sequence (CDS) of human IL-34 was synthesized and cloned into the pLenti-CMV-GFP-Puro lentiviral expression vector (GenePharma). The shRNA target sequence for human IL−34 was 5′-CAG AGC CCT CAT TGC AGT ATG-3′ and for sh-NC was 5′-GTT CTC CGA ACG TGT CAC GTT-3′. These lentiviral vectors were designed to co-express green fluorescent protein (GFP) and a puromycin resistance gene. To ensure biological reproducibility, primary FLS derived from the 6 independent RA donors (n = 6) were separately transduced. FLS were plated in 6-well plates at 1×10^6^ cells/well and transduced with lentiviral particles (1×10^8^ TU/mL, 4 µL) upon reaching 70% confluence. Following medium renewal at 24-hour intervals, infection efficiency was evaluated 48 h post-transduction through detecting green fluorescence. Stable cell lines were subsequently established via 4 µg/mL puromycin selection (Sigma-Aldrich, USA). Consequently, 6 independent stable IL-34-overexpressing FLS lines and 6 stable IL-34-knockdown FLS lines (along with their corresponding control lines) were successfully generated. All these stable cell lines were tested and utilized in the subsequent *in vitro* experiments.

### 2.5 Western blot analysis

Human synovial tissue samples from 42 RA patients and 35 control subjects, primary FLS (n = 6 biological replicates), and PBMC-derived osteoclasts (n = 6 independent donors) were lysed in RIPA lysis buffer (Solarbio, Shanghai, China). Total proteins were separated by 10% SDS-PAGE and transferred onto a polyvinylidene fluoride membrane (Millipore, USA). After blocking with 5% skimmed milk, the membranes were incubated with primary antibodies at 4°C overnight and secondary antibody at 37°C for 1 h. Protein bands were developed with the Enhanced Chemiluminescence Kit (Pierce, USA) and quantified using ImageJ software. The primary antibodies used were shown as follows: IL-34 (1:1000, ab224734), TRAP (1:1000, ab65854), NFATc1 (1:1000, ab15916), CTSK (1:1000, ab187647), MMP9 (1:1000, ab76003), p65 (1:1000, ab32536), p-p65 (1:1000, ab76032), p38 (1:1000, ab170099), p-p38 (1:1000, ab4822), ERK (1:10000, ab184699), p-ERK (1:1000, ab201015), JNK (1:10000, ab179461), p-JNK (1:1000, ab124956), GAPDH (1:2000, ab9485).

### 2.6 Immunohistochemistry

Human synovial tissues were collected from 42 RA patients and 35 control subjects. For the animal study, ankle joint tissues were harvested from mice in four groups (n = 8 per group). All tissue samples were fixed in 10% formaldehyde, paraffin-embedded, and sectioned at 4 μm thickness. Following gradient ethanol deparaffinization and rehydration, tissue sections were subjected to microwave irradiation in sodium citrate solution. Next, the slices were incubated with primary antibodies at 4°C overnight and secondary antibody for 1 h. Protein staining was conducted by DAB chromogenic reaction, and section images were examined under a light microscope (Olympus, Tokyo, Japan). Primary antibodies include: IL-34 (1:200, ab224734, Abcam), RANKL (1:200, ab169966, Abcam), CTX-I (1:100, PAA665Hu02, Cloud-Clone).

### 2.7 Cell Counting Kit-8 assay

Primary FLS (n = 6 biological replicates) were seeded into 96-well plates and incubated for 0, 24, 48, or 72 hours. At each specified time point, 10 µL of CCK-8 reagent (Dojindo, Japan) was added to individual wells for a 2 h incubation. The absorbance (450 nm) measurements were recorded using a microplate reader (BioTek, USA).

### 2.8 Transwell assay

Transwell chamber (8 μm; Corning, NY, USA) was used in Transwell assay. For the invasion assay, primary FLS (n = 6 biological replicates) were resuspended in serum-free DMEM, and 5 × 10^4^ cells were seeded into the upper compartments pre-coated with Matrigel matrix (BD Biosciences, USA). For the macrophage migration assay, THP-1-derived M0 macrophages (n = 3 independent batches) were seeded into the upper compartments without Matrigel. In both assays, DMEM supplemented with 10% FBS was added to the lower chambers. After 24-hour incubation, the cells that had moved to the lower surface of the membrane were fixed, subjected to 0.1% crystal violet staining, and quantified using a light microscope (Olympus, Tokyo, Japan).

### 2.9 Wound healing assay

Primary FLS (n = 6 biological replicates) were seeded in 6-well plates and cultured to 90-100% confluency, followed by the creation of a straight wound using a sterile 200 µL pipette tip. After PBS washing to remove debris, cells were kept in serum-free conditions for 48 h. Wound closure progression was monitored using a microscope at the initial and terminal stages, and migration quantification was examined using ImageJ software.

### 2.10 Cell apoptosis assay

Primary FLS (n = 6 biological replicates) were collected and stained with Annexin V-FITC/PI (Solarbio, Beijing, China) under light-protected conditions for 15 min, followed by apoptosis quantification through flow cytometry (BD Biosciences, USA).

### 2.11 ELISA

The concentrations of various cytokines and chemokines were measured using ELISA kits (R&D Systems, USA) according to the manufacturer’s instructions. IL-34 (Catalog #D3400) levels were determined in serum samples collected from 42 RA patients and 35 control subjects. CCL2 (Catalog #DCP00) and CXCL12 (Catalog #DSA00) were quantified in the culture supernatants of primary FLS (n = 6 biological replicates). The secretion of TNF-α (Catalog #DTA00B), IL-6 (Catalog #M6000B), and IL-1β (Catalog #MLB00C) was measured in the supernatants of THP-1-derived macrophages (n = 3 independent experiments). In the animal study, serum levels of TNF-α, IL-6, IL-1β, and IL-10 (Catalog #M1000B) were analyzed in collagen-induced arthritis (CIA) mice (four groups, n = 8 per group).

### 2.12 Flow cytometric analysis for macrophage marker detection

THP-1-derived macrophages (n = 3 independent experiments) were collected following the indicated treatment, resuspended in FACS buffer, and incubated with FITC-conjugated anti-CD68 antibody (Catalog #333805, Biolegend), together with either PE-conjugated anti-CD86 antibody (Catalog #374205, Biolegend) or PE-conjugated anti-CD206 antibody (Catalog #321105, Biolegend) for 1 h at 4°C in the dark. For the *in vivo* analysis, tumor tissues were harvested from mice in four groups (n = 8 per group), minced, and enzymatically digested to obtain single-cell suspensions. The isolated cells were stained with the same antibody panels described above. All stained samples were subsequently analyzed by flow cytometry (BD Bioscience, USA).

### 2.13 Immunofluorescence assay

THP-1-derived macrophages (n = 3 independent batches) were processed with 0.1% Triton X-100 for fixation/permeabilization. After blocking with 5% BSA, sequential incubations were performed with an iNOS primary antibody (ab178945, 1:500 dilution) at 4°C overnight and a fluorophore-conjugated secondary antibody for 2 h. Nuclei were counterstained with DAPI prior to imaging under a confocal microscopy (Zeiss, Germany).

### 2.14 Tartrate-resistant acid phosphatase staining

PBMCs (n = 6 independent donors) were seeded in 96-well plates at a density of 1×10^4^ cells/well and cultured with different CM derived from the 6 independent FLS-macrophage coculture systems, supplemented with 30 ng/mL M-CSF. After incubation for 7 and 14 days, osteoclasts were fixed with 4% paraformaldehyde for 20 min, and stained using a TRAP staining kit (Sigma-Aldrich, USA). Multinucleated TRAP-positive cells containing more than three nuclei were examined under a microscope (Olympus, Tokyo, Japan), and subsequently quantified with ImageJ software.

### 2.15 Animal experiments

Specific pathogen-free (SPF) grade male DBA/1 mice (8-week-old, 20 ± 2 g) were purchased from Zhejiang Weitong Lihua Experimental Animal Technology Co., Ltd (Zhejiang, China). Mice were maintained in a SPF facility under controlled conditions with constant temperature (22–25°C), constant humidity (50%–60%) and a 12 h light/dark cycle, and received ad libitum access to standard chow and drinking water. Experimental protocols were approved by the Laboratory Animal Nursing and Utilization Committee of Jinzhou Medical University (Approval Number: 25010401). After a 7-day acclimatization period, mice were assigned to four groups (n=8/group): control, collagen-induced arthritis (CIA) model, CIA+IgG isotype control, and CIA+anti-IL-34 treatment. CIA mice were induced as reported (32). Briefly, the bovine collagen type II (CII; Chondrex, Redmond, WA, USA) was dissolved in 0.05 mol/L acetic acid and then emulsified with equal-volume complete Freund’s adjuvant (Chondrex, USA) to prepare 1 mg/mL emulsion. CIA model was established by injecting intradermally with the collagen emulsion (100 μL) at the base of tail on day 0, followed by a booster immunization with CII/incomplete Freund’s adjuvant emulsion (100 μL) on day 21. The control animals were administrated with equivalent sterilized saline. IL-34 neutralizing antibody (catalog # MAB5195; R&D Systems, USA) and Isotype IgG were intraperitoneally injected at the dose of 10 mg/kg on day 24, 27, 30, 33, 36, 39, and 42. This dose was chosen based on previous studies in which neutralizing antibodies were used at 7.5 or 10 mg/kg in the CIA model (33, 34) and was confirmed in a preliminary dose-optimization experiment (data not shown) to provide optimal suppression of joint swelling without detectable adverse effects. All mice in each group (n = 8/group) were used for all subsequent *in vivo* assessments. The clinical arthritis scores were evaluated every 3 days after second immunization using a standardized blinded scoring system (35). The hind paw thickness was also measured to assess arthritis severity.

### 2.16 Micro-computed tomography analysis

The hind paw was fixed with 4% paraformaldehyde for 24 h and subjected to micro-CT scanning (Skyscan 1272, Bruker, Belgium) under standardized parameters: 50 kVp for voltage, 200 μA for current, and 9 μm voxel size. Three-dimensional (3D) reconstruction was generated using NRecon v1.6 software. The CT-Analyzer v1.9 software were used for analysis of 3D structural parameters, including bone mineral density (BMD, mg/cm^3^), bone volume/tissue volume (BV/TV, %), trabecular number (Tb.N), and trabecular thickness (Tb.Th, µm).

### 2.17 Histopathological evaluation

Ankle joint specimens underwent 4% paraformaldehyde fixation for 24 h followed by 3-week decalcification in 10% EDTA (pH 7.4) at 4°C. Next, paraffin-embedded tissues were sectioned into 5-μm slices for histopathological evaluation. Following standard dewaxing and hydration processes, slices were stained with hematoxylin and eosin (HE) and safranin-fast green staining both obtain from Solarbio (Beijing, China). Histopathology was blindly scored (0–4 scale) based on hyperplasia and inflammatory infiltration (33). TRAP staining (Sigma-Aldrich) was performed for osteoclast quantification using ImageJ software analysis.

### 2.18 Statistical analysis

All data are presented as the mean ± standard deviation (SD), and analyzed using SPSS 22.0. The sample size (n) for biological replicates was defined according to the experimental type: six independent samples (n = 6) were used for assays involving primary human FLS, FLS-macrophage coculture systems, and subsequent PBMC-derived osteoclastogenesis; three independent experiments (n = 3) were conducted for those using the THP-1 cell line; and eight mice per group (n = 8) were included in the *in vivo* animal studies. For experiments with larger sample sizes (n = 6 and 8), data normality and homogeneity of variance were formally assessed using the Shapiro-Wilk test and Levene’s test, respectively (no significant deviations were found, *P* > 0.05), to ensure that the assumptions for parametric tests were met. For small sample size experiments (n = 3), formal normality testing lacks sufficient statistical power; therefore, normal distribution and equal variance were assumed based on standard empirical practices for continuous data in basic molecular biology. The Student’s *t*-test was employed for comparing two groups, whereas one-way ANOVA was applied for evaluating multiple groups, followed by Tukey’s *post hoc* test for multiple comparisons correction. For comparisons of baseline characteristics between RA and control groups, an independent t-test was used for continuous variables (age), and the chi-square test was applied for categorical variables (sex, hypertension, diabetes). *P* < 0.05 was regarded as statistically significant.

## 3 Results

### 3.1 IL-34 expression was upregulated in clinical RA samples and positively correlated with the severity of bone erosion

**Figure 1:**
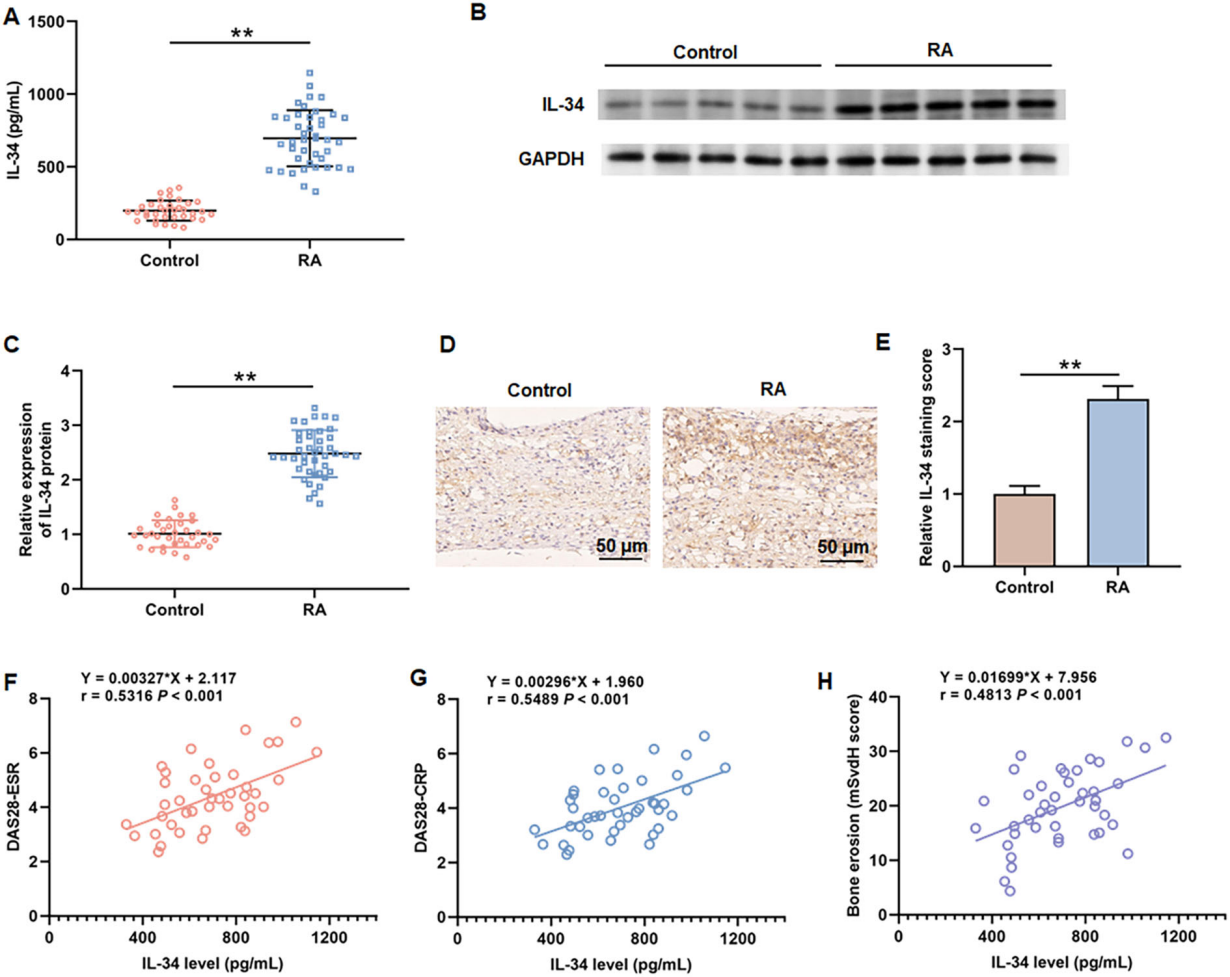
IL-34 expression was upregulated in clinical RA samples and positively correlated with the severity of bone erosion. RA patients (n=42, male = 15, female = 27, the average age = 51.9 ± 10.3) and control patients undergoing joint surgery (n=35, male = 14, female = 21, the average age = 47.8 ± 12.2) were recruited. **(A)** Serum IL-34 levels were examined using an ELISA kit. **(B, C)** Western blot analysis and **(D, E)** IHC was employed to evaluate IL-34 protein levels in the synovial tissues of all participates (n = 42 RA and n = 35 control). **(F–H)** Correlation between IL-34 expression and DAS28-ESR (r = 0.28260.5316, *P* < 0.001), DAS28-CRP (r = 0.54890.3013, *P* < 0.001), and bone erosion score (r = 0.4813, *P* < 0.001) in RA patients (n = 42). Data were presented as mean ± SD. **P < 0.01.

Clinical investigations were conducted to examine IL-34 expression profiles in RA patients (n = 42) and control subjects (n = 35). Initial analysis implicated that serum IL-34 levels in RA patients were upregulated compared to normal controls (**Figure 1A**). This differential expression pattern was further confirmed in RA synovial tissues. Western blot results demonstrated a significant upregulation of IL-34 protein in RA synovial tissues (**Figures 1B, C**), while IHC results provided complementary spatial localization evidence of enhanced IL-34 expression in RA synovial tissues (**Figures 1D, E**). Subsequent clinical parameter correlation studies (n = 42 RA patients) identified that serum IL-34 concentration was positively correlated with the disease activity reflected by the DAS28-ESR (r = 0.5316, *P* < 0.001) (**Figure 1F**) and DAS28-CRP (r = 0.5489, *P* < 0.001) (**Figure 1G**). Notably, a positive correlation was also observed between serum IL-34 levels and bone erosion score (r = 0.4813, *P* < 0.001) (**Figure 1H**). These moderate correlations support the hypothesis that IL-34 may contribute to pathological bone erosion mechanisms underlying RA pathogenesis.

### 3.2 IL-34 overexpression facilitated proliferation, migration and invasion and inhibited apoptosis in FLS

**Figure 2:**
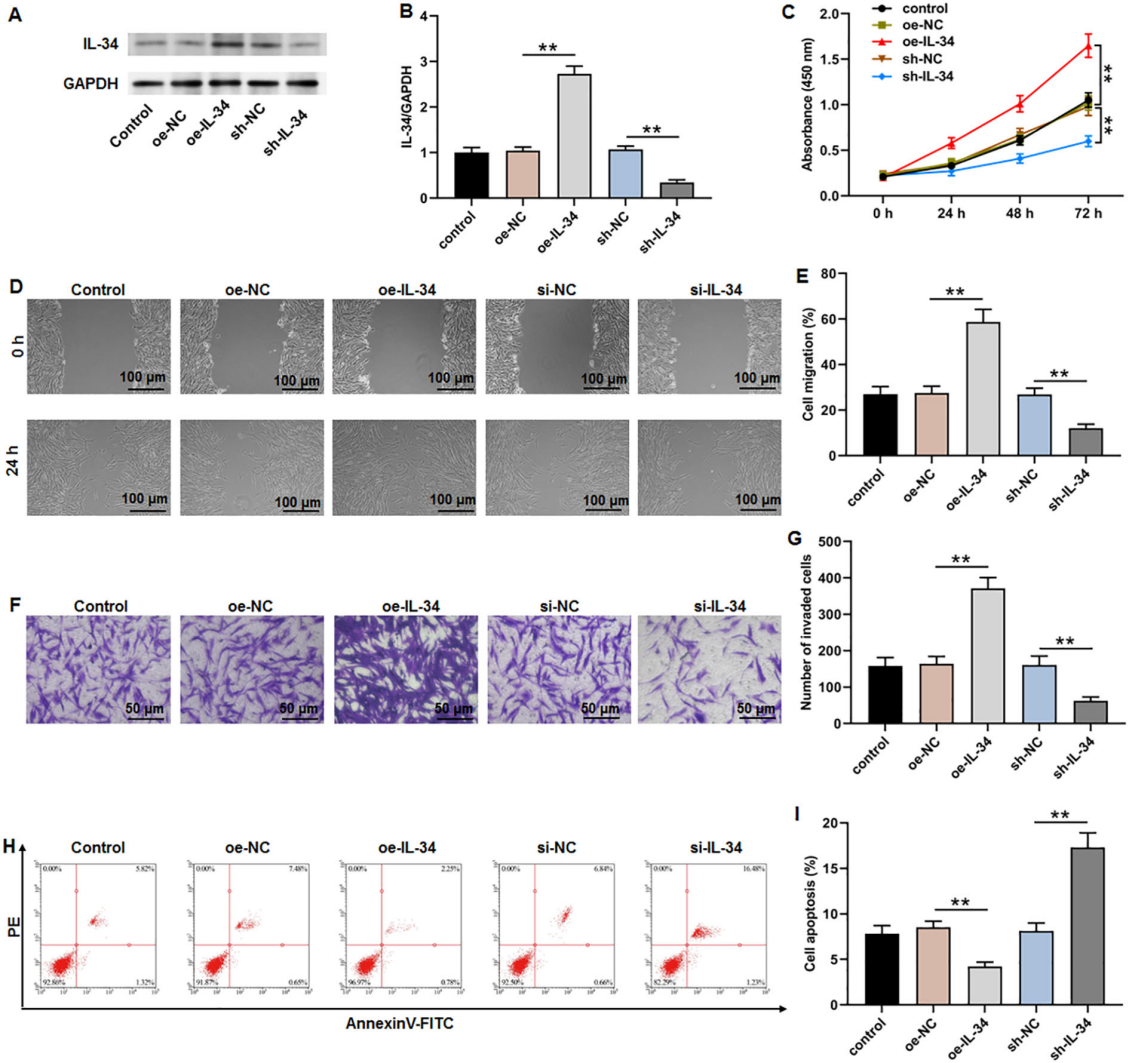
IL-34 overexpression facilitated proliferation, migration and invasion and inhibited apoptosis in FLS. **(A, B)** IL-34 protein expression was quantified by Western blot analysis. **(C)** Cell proliferation was assessed by CCK-8 assay. **(D, E)** Cell migration was examined by wound healing assay. **(F, G)** Cell invasion was quantified by Transwell assay. **(H, I)** Cell apoptosis was analyzed by flow cytometry. All experiments were performed using primary FLS (n = 6 biological replicates). Data were presented as mean ± SD. **P < 0.01.

To elucidate the functional significance of IL-34 in FLS, gain- and loss-of-function experiments were employed using primary FLS (n = 6 biological replicates) through transfection with IL-34 overexpression plasmids or shRNA. Western blot analysis suggested that oe-IL-34 transfection upregulated IL-34 protein expression, while sh-IL-34 transfection downregulated IL-34 protein expression in FLS (**Figures 2A, B**). IL-34 overexpression enhanced cell proliferation (**Figure 2C**), migration (**Figures 2D, E**) and invasion (**Figures 2F, G**) in FLS. In contrast, IL-34 silencing exerted inhibitory effects on these aggressive cellular phenotypes. Furthermore, IL-34 overexpression inhibited apoptosis, while IL-34 silencing showed the opposite results (**Figures 2H, I**).

### 3.3 The CM from IL-34-overexpressing FLS promoted macrophage recruitment and M1 polarization

**Figure 3:**
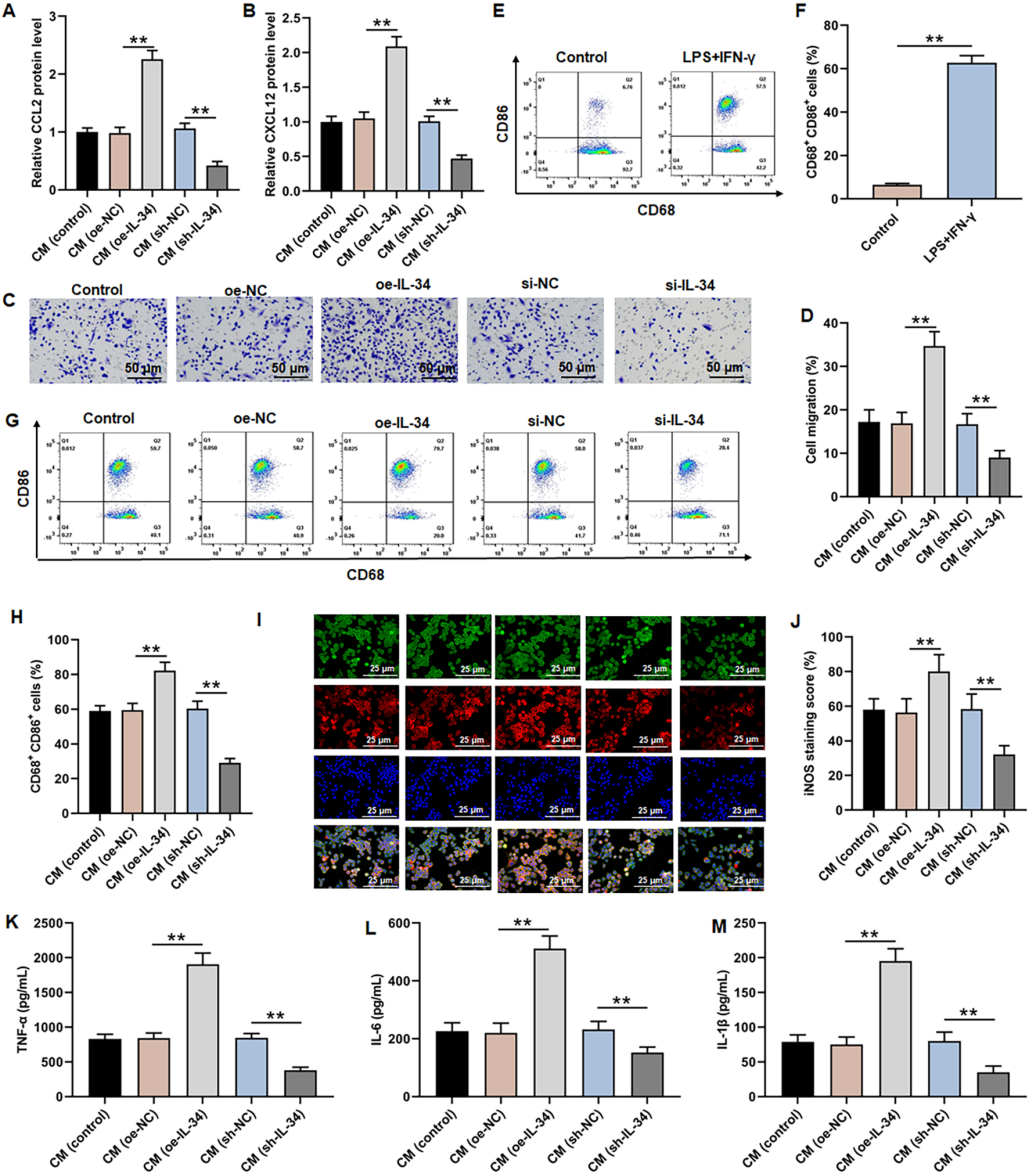
The CM from IL-34-overexpressing FLS promoted macrophage recruitment and M1 polarization. **(A, B)** CCL2 and CXCL12 levels in the CM of primary FLS (n = 6) were examined by ELISA kits. **(C, D)** THP-1-derived M0 macrophage migration was quantified by Transwell assay (n = 3 independent experiments). **(E, F)** THP-1 cell-differentiated macrophages (n = 3) were treated with 100 ng/mL LPS and 20 ng/mL IFN-γ to induce M1 macrophage polarization, and the percentages of CD68^+^CD86^+^-positive macrophages were assessed by flow cytometry. **(G, H)** M1 macrophages (n = 3) were incubated with CM from IL-34-manipulated FLS, and the percentages of CD68^+^CD86^+^ macrophages were assessed by flow cytometry. **(I, J)** Immunofluorescence staining was utilized to evaluate iNOS expression (n = 3). **(K)** TNF-α, **(L)** IL-6, and **(M)** IL-1β were examined by ELISA kits (n = 3). Data were presented as mean ± SD. **P < 0.01.

To further clarify the role of IL-34 in mediating FLS-macrophage crosstalk underlying RA pathogenesis, we investigated its regulatory effects on chemokine-driven macrophage recruitment and polarization. Specifically, IL-34 overexpression in FLS (n = 6) significantly upregulated the secretion of CCL2 and CXCL12 functioned as key chemokines responsible for macrophage recruitment, whereas IL-34 silencing suppressed their secretion (**Figures 3A, B**). Notably, the CM from IL-34-overexpressing FLS promoted the migration of THP-1-derived M0 macrophages (n = 3 independent experiments), whereas CM from IL-34-silenced FLS inhibited macrophage migration (**Figures 3C, D**). Then, THP-1 cell-differentiated macrophages (n = 3) were induced into M1 polarization, followed by incubation with CM from IL-34-manipulated FLS. Flow cytometry analysis confirmed a significant increase in CD68^+^CD86^+^ M1 macrophages after LPS/IFN-γ stimulation (**Figures 3E, F**). Notably, CM from IL-34-overexpressing FLS further increased the M1 population, while CM from IL-34-knockdown FLS reduced this proportion (**Figures 3G, H**). Consistent with these findings, immunofluorescence and ELISA results illustrated that CM from IL-34-overexpressing FLS increased the expression of M1 macrophage markers, including iNOS (**Figures 3I, J**), TNF-α (**Figure 3K**), IL-6 (**Figure 3L**), and IL-1β (**Figure 3M**), whereas CM from IL-34-silenced FLS showed the opposing effects.

### 3.4 IL-34-mediated FLS-macrophage crosstalk accelerated osteoclastogenesis

**Figure 4:**
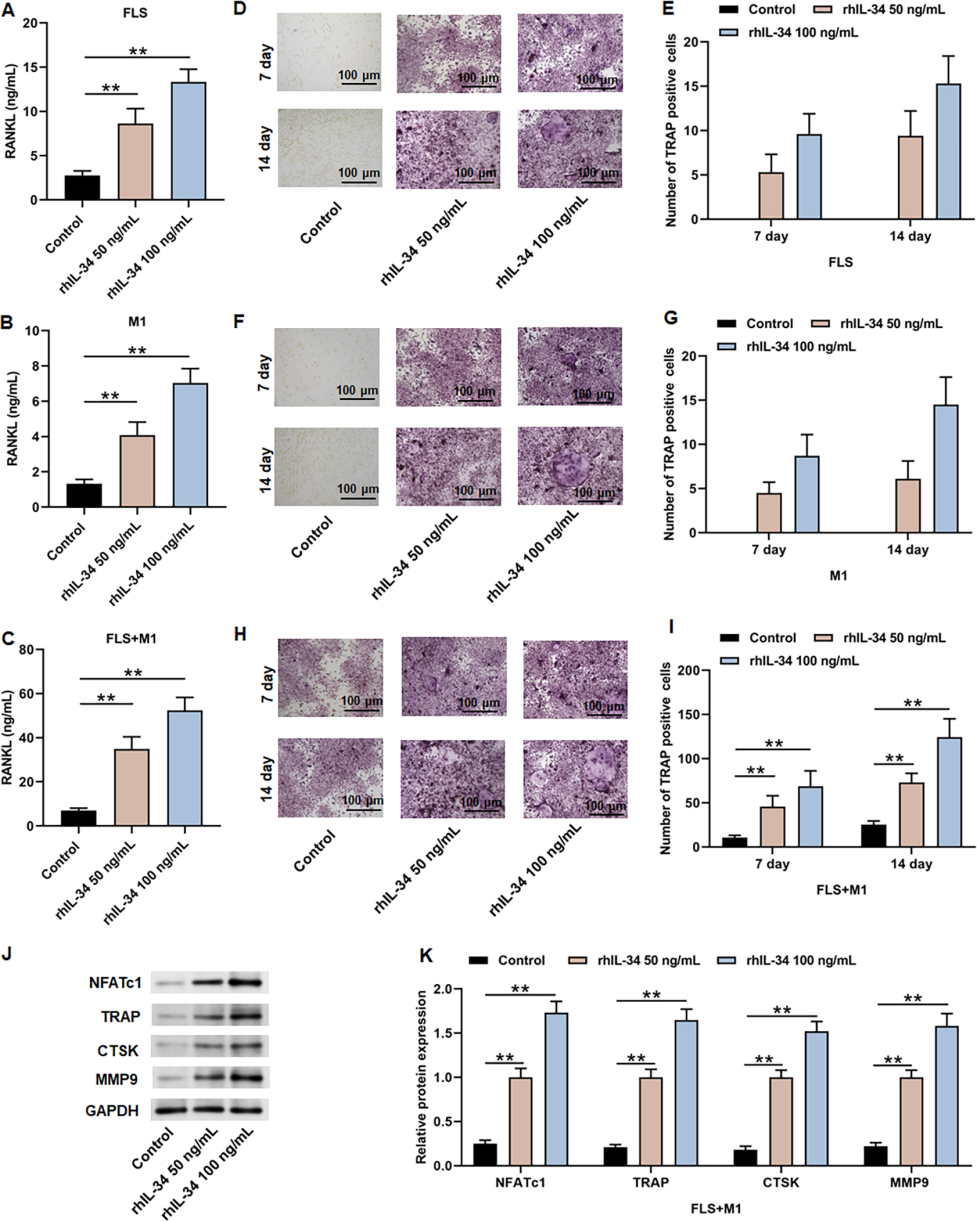
IL-34-mediated FLS-macrophage crosstalk accelerated osteoclastogenesis. **(A–C)** Primary FLS (n = 6), M1 macrophages (n = 3 independent differentiation batches) were used to establish 6 independent coculture sets. Specifically, each independent macrophage batch was paired with FLS from two distinct RA donors. These coculture systems were respectively treated with rhIL-34 at 50 or 100 ng/mL for 72 h. RANKL levels in the unpooled, donor-specific CM were examined by ELISA kits. **(D–I)** PBMCs (n = 6 independent donors) were incubated with the matched, donor-specific CM from these groups for 7 and 14 days in a one-to-one manner (one CM set per PBMC donor). TRAP staining was employed to assess osteoclastogenesis. **(J, K)** NFATc1, TRAP, CTSK and MMP9 protein levels were examined by Western blot analysis after incubation with the 6 independent coculture-derived CM samples for 14 days. Data were presented as mean ± SD. **P < 0.01.

To assess the osteoclastogenic potential of IL-34-mediated FLS-macrophage crosstalk, primary FLS (n = 6), M1 macrophages (n = 3), and their coculture system (n = 6 independent sets) were respectively treated with rhIL-34 at 50 or 100 ng/mL for 72 h. Analysis of CM revealed a dose-dependent elevation in RANKL levels across all groups (**Figures 4A–C**). Notably, rhIL-34-stimulated coculture systems exhibited markedly higher RANKL concentrations (34.90 and 52.46 ng/mL) compared to FLS (8.64 and 13.33 ng/mL) or M1 macrophages (4.08 and 7.04 ng/mL) alone. To further assess osteoclast differentiation, PBMCs (n = 6 independent donors) were incubated with CM from these groups. After incubation for 7 and 14 days, TRAP staining suggested minimal osteoclast formation in PBMCs exposed to CM from individual cell cultures (**Figures 4D–G**). In contrast, coculture-derived CM significantly increased TRAP-positive osteoclasts by Day 7, with further increases by Day 14 (**Figures 4H, I**). Consistently, we showed that osteoclast-related markers including NFATc1, TRAP, CTSK and MMP9 were increased after incubation with coculture-derived CM for 14 days (**Figures 4J, K**). These collective results demonstrated that IL-34-mediated FLS-macrophage crosstalk accelerated osteoclastogenesis.

### 3.5 RANKL inhibitor abrogated the promoting effect of IL-34-mediated FLS-macrophage crosstalk on osteoclastogenesis

**Figure 5:**
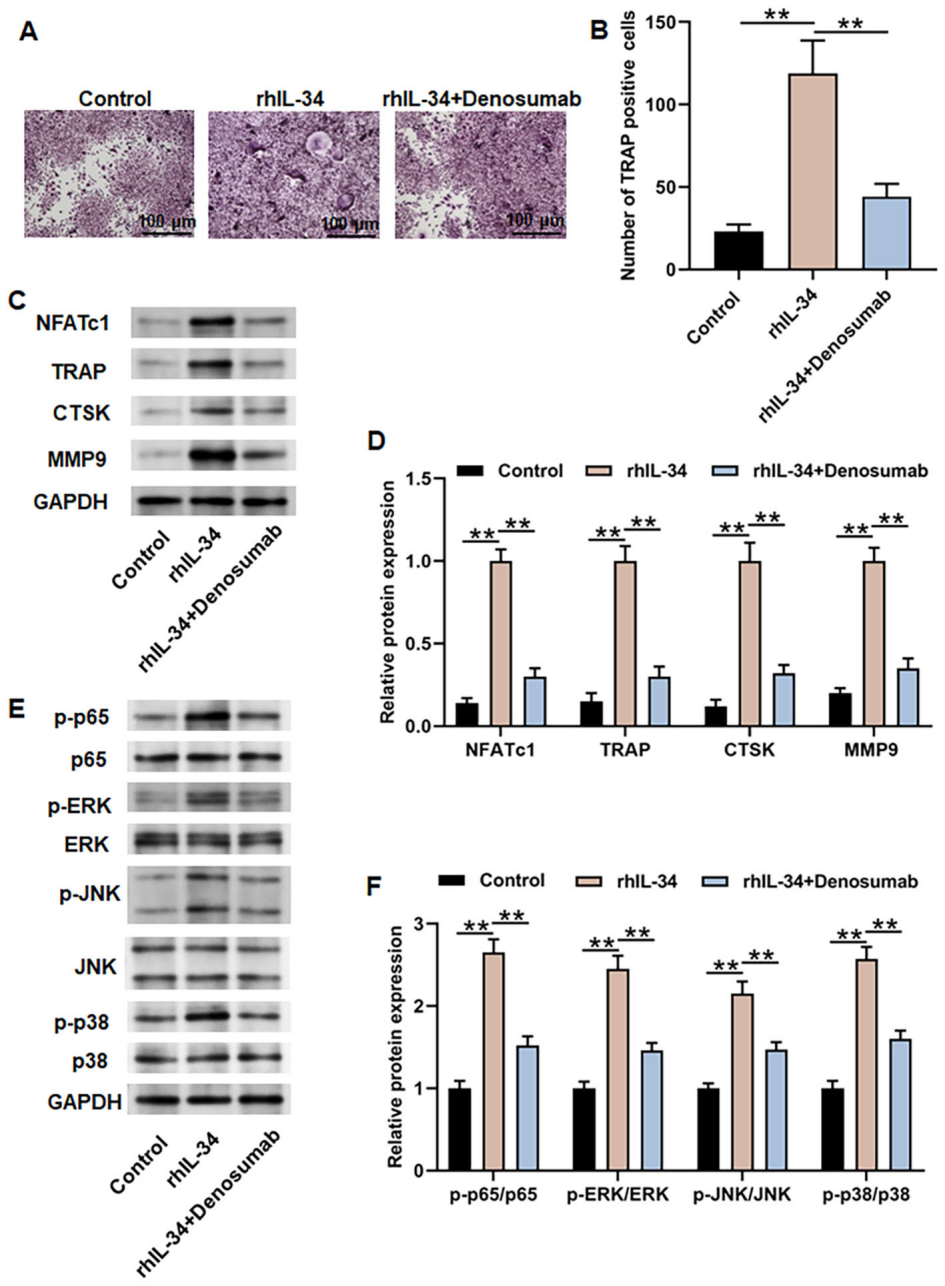
RANKL inhibitor abrogated the promoting effect of IL-34-mediated FLS-macrophage crosstalk on osteoclastogenesis. PBMCs (n = 6) were exposed to donor-matched CM from 100 ng/mL rhIL-34-treated coculture with or without RANKL inhibitor denosumab (10 μg/mL). Each of the 6 replicates represents an independent pairing of a specific coculture CM set and a unique PBMC donor. **(A, B)** TRAP staining was employed to assess the number of osteoclasts. **(C, D)** NFATc1, TRAP, CTSK and MMP9 protein levels were examined by Western blot analysis. **(E, F)** The phosphorylated levels of NF-κB p65, ERK, JNK, and p38 were examined by Western blot analysis. Data were presented as mean ± SD. **P < 0.01.

To delineate the role of RANKL in IL-34-driven osteoclastogenesis, PBMCs (n = 6 independent donors) were exposed to CM from 100 ng/mL rhIL-34-treated coculture with or without RANKL inhibitor denosumab (10 μg/mL). TRAP staining implicated that TRAP-positive osteoclasts was markedly increased in the rhIL-34-CM group, which were effectively abrogated by denosumab treatment (**Figures 5A, B**). Moreover, rhIL-34-CM treatment upregulated the protein levels of NFATc1, TRAP, CTSK and MMP9, while denosumab counteracted these effects (**Figures 5C, D**). Mechanistically, rhIL-34-CM activated the downstream pathways of RANKL-RANK signaling, evidenced by increased phosphorylation of NF-κB p65 and MAPK pathway members (ERK, JNK, p38) (**Figures 5E, F**). However, denosumab treatment attenuated their expression. Importantly, denosumab attenuated these phosphorylation events, confirming RANKL dependency. Collectively, we revealed that IL-34-mediated FLS-macrophage crosstalk accelerated osteoclastogenesis by activating RANKL-RANK signaling and its downstream NF-κB and MAPK signaling cascades.

### 3.6 IL-34 neutralizing antibody attenuated RA severity in CIA mice

**Figure 6:**
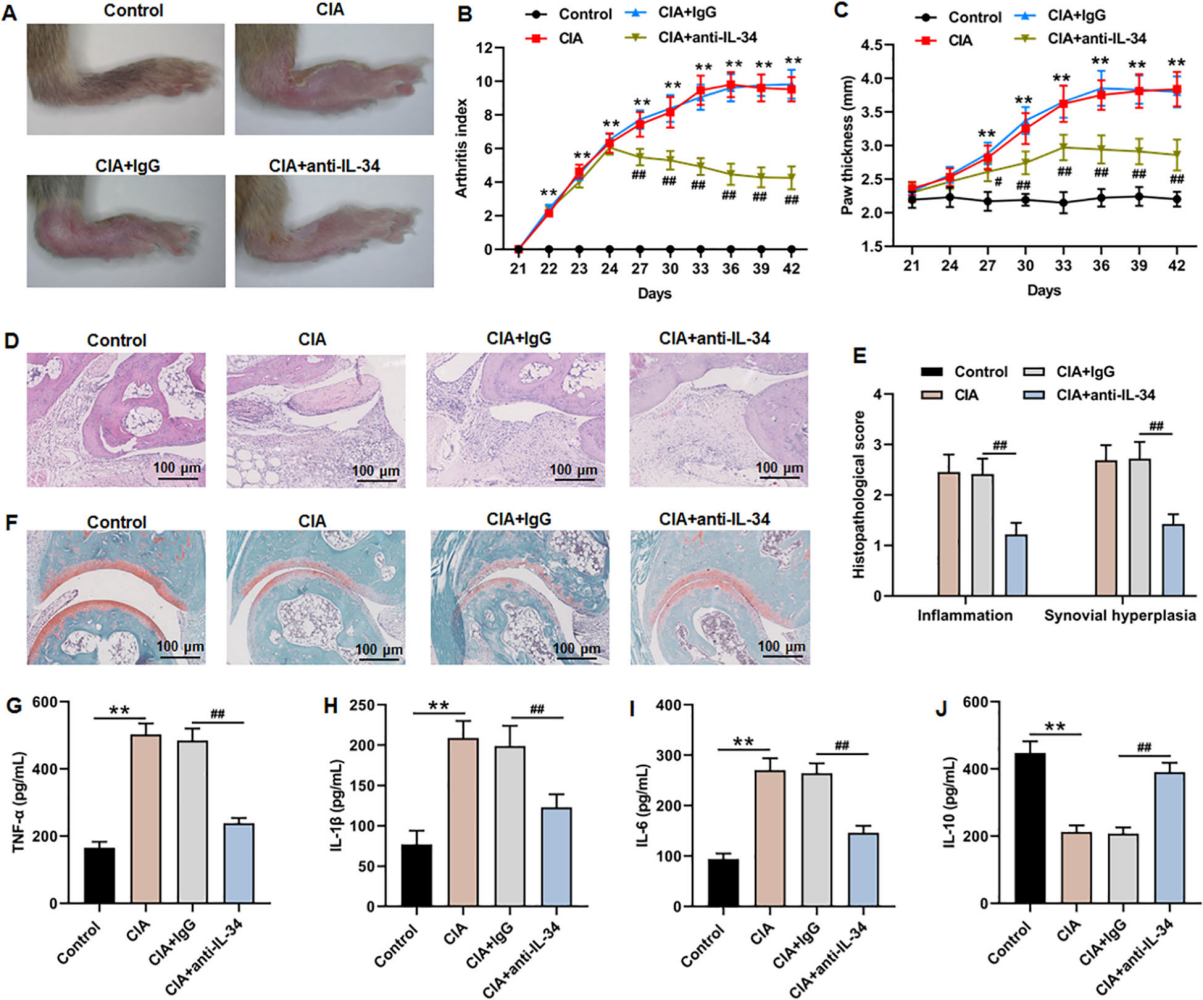
IL-34 neutralizing antibody alleviated RA symptoms in CIA mice. CIA mouse model was established and treated with IL-34 neutralizing antibody or IgG control (four groups, n=8 per group). **(A)** Representative photographs of paws from all groups after finishing treatment. **(B)** The arthritis score and **(C)** paw thickness of mice in each group were evaluated every 3 days after second immunization. Histopathological analysis of ankle joint sections through **(D, E)** HE staining and **(F)** safranin O-fast green. **(G-J)** Serum TNF-α, IL-6, IL-1β and IL-10 levels were examined by ELISA kits. Data were presented as mean ± SD. **P < 0.01, compared with control group; ##*P* < 0.01, compared with the CIA+IgG group.

To elucidate the therapeutic effects of IL-34 blockade on RA progression, a CIA mouse model was established and treated with IL-34 neutralizing antibody or IgG control (four groups, n = 8 mice per group). The model group developed characteristic arthritic features including pronounced erythema and edema in hind paw joints (**Figure 6A**), which were substantially attenuated after IL-34 neutralizing antibody intervention. The arthritis score (**Figure 6B**) and paw swelling (**Figure 6C**) were continuously increased after second immunization, while these two indicators were significantly decreased after IL-34 neutralizing antibody treatment. Histopathological analysis of ankle joint sections showed that CIA mice exhibited severe synovial hyperplasia and inflammatory infiltration (**Figures 6D, E**), and extensive cartilage destruction (**Figure 6F**), all of which were remarkably attenuated in antibody-treated animals. Additionally, the serum levels of TNF-α, IL-6 and IL-1β were significantly increased and IL-10 level was decreased in CIA mice, which were abrogated by anti-IL-34 antibody treatment (**Figures 6G–J**). These collective findings suggested that IL-34 neutralizing antibody attenuated RA severity in CIA mice.

### 3.7 IL-34 neutralizing antibody mitigated bone erosion in CIA mice

**Figure 7:**
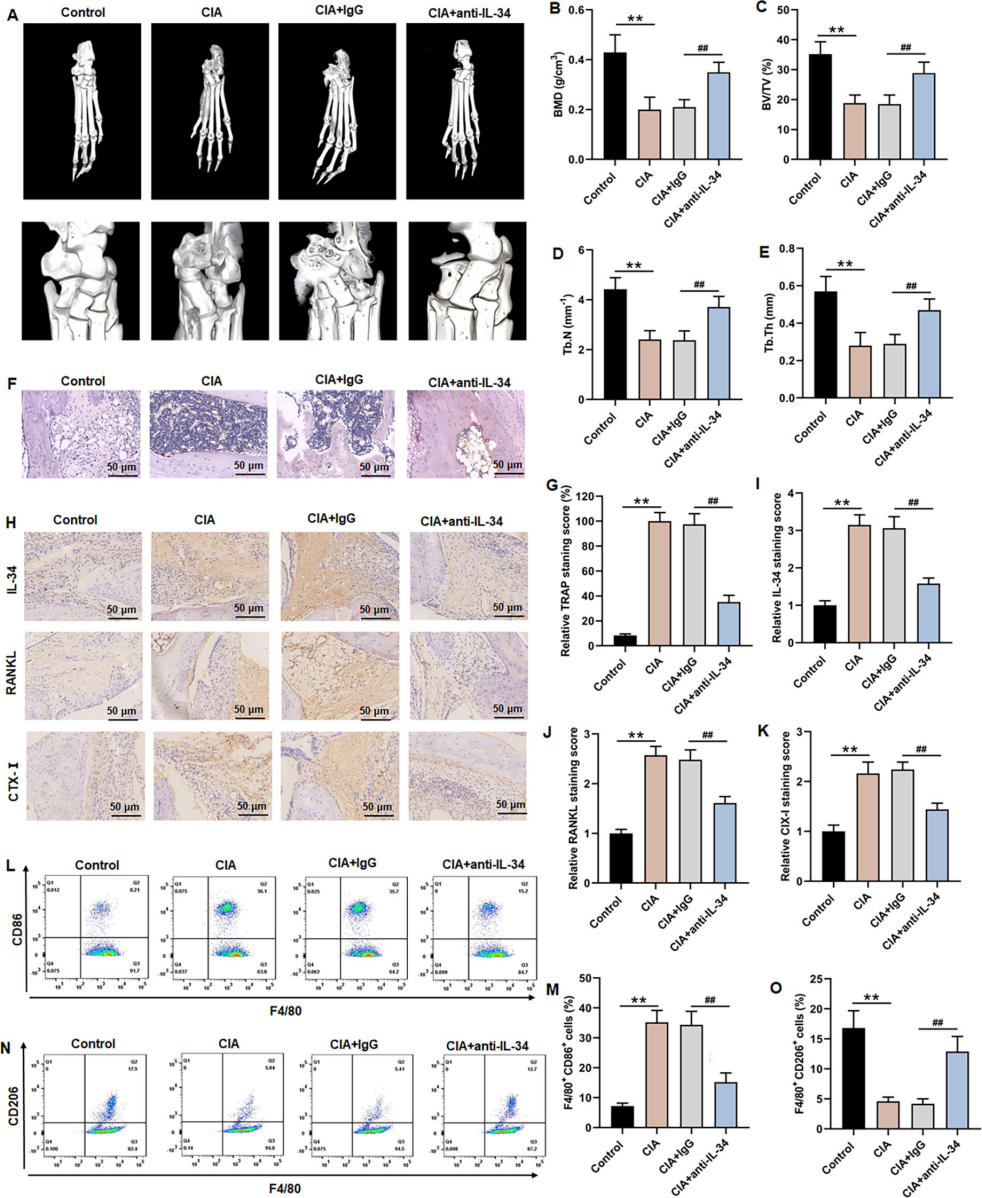
IL-34 neutralizing antibody mitigated bone erosion in CIA mice. **(A)** Representative images of 3D reconstructed bone morphology of ankle joints in Micro-CT analysis. Quantitative analysis of **(B)** BMD, **(C)** BV/TV, **(D)** Tb.N, and **(E)** Tb.Th. **(F, G)** Osteoclastogenesis in ankle joints was evaluated by TRAP staining. **(H–K)** IL-34, RANKL, and CTX-I levels in the ankle joints were examined by ELISA kits. **(L–O)** The percentages of F480^+^CD86^+^-positive M1 macrophages and F480^+^CD206^+^-positive M2 macrophages were assessed by flow cytometry. n = 8 per group. Data were presented as mean ± SD. **P < 0.01, compared with control group; ##*P* < 0.01, compared with the CIA+IgG group.

We further investigate the effects of IL-34 neutralizing antibody intervention on bone erosion in CIA mice (n = 8 per group). 3D reconstructed Micro-CT images revealed pronounced bone destruction in CIA mice, featuring irregular bone surfaces, trabecular porosity, and extensive cortical erosion at ankle joints (**Figure 7A**), while anti-IL-34 intervention substantially ameliorated these structural deteriorations. Quantitative analysis indicated that CIA-induced reductions in BMD (**Figure 7B**), BV/TV (**Figure 7C**), Tb.N (**Figure 7D**), and Tb.Th (**Figure 7E**) were significantly restored by IL-34 neutralizing antibody treatment. TRAP staining further revealed that IL-34 neutralization suppressed osteoclastogenesis in ankle joints (**Figures 7F, G**). Furthermore, we confirmed the increased levels of IL-34, RANKL, and a bone resorption marker cross-linked C-telopeptide of type I collagen (CTX-I) in the ankle joints of CIA mice (**Figures 7H–K**), which were inhibited by IL-34 neutralizing antibody treatment. Additionally, flow cytometry analysis demonstrated that IL-34 neutralizing antibody significantly inhibited M1 macrophages in CIA mice (**Figures 7L–O**). These multimodal findings highlight that IL-34 blockade as a treatment method for alleviating bone erosion in RA.

## 4 Discussion

The present study identifies IL-34 as a crucial regulator of the pathogenic crosstalk between FLS and macrophages in RA, driving both inflammatory amplification and osteoclast-mediated bone erosion. Our findings highlight IL-34 as a critical molecule linking synovial hyperplasia, macrophage dysregulation, and osteoclastogenesis-associated bone erosion in RA, offering novel insights into RA pathophysiology and potential therapeutic targeting.

We demonstrated that IL-34 is upregulated in RA serum and synovial tissues, correlating with disease activity (DAS28-ESR/CRP) and bone erosion severity. This is consistent with the previously reported findings (20–22). Previous studies have demonstrated that IL-34 facilitates FLS proliferation and suppresses apoptosis (23, 24), and our study produced similar results. Notably, our data extend these findings by mechanistically connecting IL-34 to the crosstalk between FLS and macrophages within the RA microenvironment. Crucially, IL-34 overexpression increased the secretion of chemokines in primary FLS, thus triggering macrophage recruitment and infiltration. Furthermore, incubation with CM from IL-34-overexpressing FLS induced macrophage M1 polarization and inflammatory cytokine secretion. This aligns with the recognized role of FLS as “non-immune immune cells” in shaping the RA microenvironment (36), but our results uniquely highlight IL-34 as a crucial mediator of this process.

Additionally, we uncovered that IL-34-mediated FLS-macrophage crosstalk directly accelerates osteoclastogenesis through a RANKL-dependent mechanism. RANKL binds to its receptor RANK on the surface of osteoclast precursors to activate a series of signaling cascades, such as the NF-κB and MAPK pathways, subsequently inducing the transcriptional expression of NFATc1 (37). The self-amplification mechanism of NFATc1 and its cooperation with transcription factors form a network to drive the expression of osteoclast-specific genes like TRAP and CTSK, accurately regulating bone resorption (38). Our experiments revealed that IL-34 stimulation in FLS-macrophage conculture system significantly upregulated RANKL production. This elevated RANKL secretion subsequently activated RANKL-RANK signaling and downstream NF-κB and MAPK pathways in osteoclast precursors, further accelerating osteoclastogenesis. However, denosumab abrogated these effects through its high-affinity binding to RANKL, thereby competitively inhibiting its interaction with RANK on the surface of osteoclast precursors. This RANKL-dependent mechanism explains the positive correlation between IL-34 levels and bone erosion in RA patients (22). Notably, IL-34 exhibits functional similarities to M-CSF in promoting osteoclastogenesis via CSF-1R binding (19). However, we emphasize its unique ability to simultaneously target FLS and macrophages, forming a self-reinforcing vicious feedback loop within the RA microenvironment. Unlike M-CSF, the pleiotropic effects of IL-34 on FLS proliferation, inflammatory macrophage polarization, and osteoclastogenesis make it a multifaceted contributor to the vicious cycle of RA inflammation and tissue destruction.

IL-34 neutralization in CIA mice alleviated characteristic RA symptoms including pronounced erythema and swelling in the hind paw joints. Moreover, IL-34 inhibition attenuated synovial hyperplasia, M1 macrophage polarization, inflammatory infiltration, and cartilage destruction in CIA mice, indicating the pleiotropic efficacy of IL-34 inhibition in RA. Zhang et al. proposed that rh-IL-34 injection aggravated the severity of RA in CIA mice (39), which indirectly supports our results. Our in-depth studies demonstrated that IL-34 neutralizing antibody mitigated bone erosion and preserved bone integrity in CIA mice as reflected by the rescue of BMD, BV/TV, Tb.N, and Tb.Th metrics. Mechanistically, IL-34 neutralization suppressed RANKL expression and osteoclastogenesis in ankle joints. Importantly, denosumab abrogated IL-34-driven osteoclastogenesis, suggesting RANKL as a key therapeutic target. However, targeting IL-34 may provide broader benefits by disrupting multiple upstream pathogenic events. Compared to TNF-α inhibitors, which primarily target the inflammation cascades and bone destruction (40), IL-34 blockade could synergistically inhibit FLS-macrophage crosstalk and osteoclastogenesis. Nevertheless, future studies should explore localized delivery strategies to minimize the potential off-target effects of systemic IL-34 inhibition.

This investigation still has several limitations. First, the limited cohort size of RA patients may affect the statistical results, and larger-scale validation is needed to ensure broader generalizability. Second, due to the limited cohort size of RA patients (n=42) and incomplete clinical records regarding specific biologic therapies, we were unable to conduct a robust multivariate analysis to adjust for potential confounders such as age, sex, and medication regimens. Therefore, larger-scale clinical validations with comprehensive multivariate adjustments are needed to ensure broader applicability. Third, although the CIA-induced RA mouse model provides valuable mechanistic insights, it may not fully recapitulate the complexity of human RA pathophysiology. Additionally, the precise upstream regulators of IL-34 in FLS remain to be investigated.

Taken together, our findings highlight IL-34 as a crucial regulator of FLS-macrophage crosstalk in the RA immune microenvironment. IL-34-mediated FLS-macrophage crosstalk drives bone erosion in RA through RANKL-dependent osteoclastogenesis. We suggest that IL-34 blockade is a promising therapeutic method for concurrently alleviating synovial hyperplasia, inflammation and bone erosion in RA.

## Data Availability

The original contributions presented in the study are included in the article/**Supplementary Material**. Further inquiries can be directed to the corresponding author.

## References

[1] Radu AF Bungau SG . Management of rheumatoid arthritis: an overview. Cells. (2021) 10:2857. doi: 10.3390/cells10112857 34831081 PMC8616326

[2] Fu Q Gao Y Zhao H Wang Z Wang J . Galangin protects human rheumatoid arthritis fibroblast-like synoviocytes via suppression of the NF-κB/NLRP3 pathway. Mol Med Rep. (2018) 18:3619–24. doi: 10.3892/mmr.2018.9422 30152847

[3] Huang J Fu X Chen X Li Z Huang Y Liang C . Promising therapeutic targets for treatment of rheumatoid arthritis. Front Immunol. (2021) 12:686155. doi: 10.3389/fimmu.2021.686155 34305919 PMC8299711

[4] Venetsanopoulou AI Alamanos Y Voulgari PV Drosos AA . Epidemiology of rheumatoid arthritis: genetic and environmental influences. Expert Rev Clin Immunol. (2022) 18:923–31. doi: 10.1080/1744666x.2022.2106970 35904251

[5] Scherer HU Häupl T Burmester GR . The etiology of rheumatoid arthritis. J Autoimmun. (2020) 110:102400. doi: 10.1016/j.jaut.2019.102400 31980337

[6] Jang S Kwon EJ Lee JJ . Rheumatoid arthritis: pathogenic roles of diverse immune cells. Int J Mol Sci. (2022) 23:905. doi: 10.3390/ijms23020905 35055087 PMC8780115

[7] Kay J Upchurch KS . ACR/EULAR 2010 rheumatoid arthritis classification criteria. Rheumatol (Oxford). (2012) 51:vi5–9. doi: 10.1093/rheumatology/kes279 23221588

[8] Li Y Zhang J An X Li Y . Evaluation of carotid artery elastic function using ultrafast pulse wave velocity in patients with rheumatoid arthritis. Echocardiography. (2022) 39:552–60. doi: 10.1111/echo.15325 35212028

[9] Tsaltskan V Firestein GS . Targeting fibroblast-like synoviocytes in rheumatoid arthritis. Curr Opin Pharmacol. (2022) 67:102304. doi: 10.1016/j.coph.2022.102304 36228471 PMC9942784

[10] Nygaard G Firestein GS . Restoring synovial homeostasis in rheumatoid arthritis by targeting fibroblast-like synoviocytes. Nat Rev Rheumatol. (2020) 16:316–33. doi: 10.1038/s41584-020-0413-5 32393826 PMC7987137

[11] Lin S Zhou Z Xu C Zeng F Shi Z Sun J . Cytokine regulation and fast inflammation resolution in early rheumatoid arthritis by cerium-modified gold nanoclusters. ACS Appl Mater Interfaces. (2022) 14:18053–63. doi: 10.1021/acsami.1c22831 35417127

[12] Chang L Feng X Gao W . Proliferation of rheumatoid arthritis fibroblast-like synoviocytes is enhanced by IL-17-mediated autophagy through STAT3 activation. Connect Tissue Res. (2019) 60:358–66. doi: 10.1080/03008207.2018.1552266 30477351

[13] Rana AK Li Y Dang Q Yang F . Monocytes in rheumatoid arthritis: circulating precursors of macrophages and osteoclasts and, their heterogeneity and plasticity role in RA pathogenesis. Int Immunopharmacol. (2018) 65:348–59. doi: 10.1016/j.intimp.2018.10.016 30366278

[14] Liu X Zhang H Chang X Shen J Zheng W Xu Y . Upregulated expression of CCR3 in rheumatoid arthritis and CCR3-dependent activation of fibroblast-like synoviocytes. Cell Biol Toxicol. (2017) 33:15–26. doi: 10.1007/s10565-016-9356-7 27495116

[15] Cutolo M Campitiello R Gotelli E Soldano S . The role of M1/M2 macrophage polarization in rheumatoid arthritis synovitis. Front Immunol. (2022) 13:867260. doi: 10.3389/fimmu.2022.867260 35663975 PMC9161083

[16] Zheng Y Wei K Jiang P Zhao J Shan Y Shi Y . Macrophage polarization in rheumatoid arthritis: signaling pathways, metabolic reprogramming, and crosstalk with synovial fibroblasts. Front Immunol. (2024) 15:1394108. doi: 10.3389/fimmu.2024.1394108 38799455 PMC11116671

[17] Matsuda K Shiba N Hiraoka K . New insights into the role of synovial fibroblasts leading to joint destruction in rheumatoid arthritis. Int J Mol Sci. (2023) 24:5173. doi: 10.3390/ijms24065173 36982247 PMC10049180

[18] Yasuda H . Discovery of the RANKL/RANK/OPG system. J Bone Miner Metab. (2021) 39:2–11. doi: 10.1007/s00774-020-01175-1 33389131

[19] Zhou RP Wu XS Xie YY Dai BB Hu W Ge JF . Functions of interleukin-34 and its emerging association with rheumatoid arthritis. Immunology. (2016) 149:362–73. doi: 10.1111/imm.12660 27550090 PMC5095491

[20] Zhang F Ding R Li P Ma C Song D Wang X . Interleukin-34 in rheumatoid arthritis: potential role in clinical therapy. Int J Clin Exp Med. (2015) 8:7809–15. PMC4509278 26221333

[21] Wang B Ma Z Wang M Sun X Tang Y Li M . IL-34 upregulated Th17 production through increased IL-6 expression by rheumatoid fibroblast-like synoviocytes. Mediators Inflammation. (2017) 2017:1567120. doi: 10.1155/2017/1567120 28659662 PMC5474253

[22] Li N Jiang L Cai Y Liu JY Zhao T Kong N . The correlation between interleukin-34 and bone erosion under ultrasound in rheumatoid arthritis. Mod Rheumatol. (2020) 30:269–75. doi: 10.1080/14397595.2019.1593576 30880555

[23] Elkhider A Wei J Al-Azab M Tang Y Walana W Li W . IL-34 modulates rheumatoid synovial fibroblasts proliferation and migration via ERK/AKT signalling pathway. Clin Exp Rheumatol. (2020) 38:479–87. 31498070

[24] Li X Lei Y Gao Z Wu G Gao W Xia L . IL-34 affects fibroblast-like synoviocyte proliferation, apoptosis and function by regulating IL-17. Sci Rep. (2021) 11:16378. doi: 10.1038/s41598-021-95839-1 34385542 PMC8361173

[25] Yang H Luo Y Lai X . Il-34 regulates MAPKs, PI3K/Akt, JAK and NF-κB pathways and induces the expression of inflammatory factors in RA-FLS. Clin Exp Rheumatol. (2022) 40:1779–88. doi: 10.55563/clinexprheumatol/6t1d4i 35200127

[26] Wang B Tang Y Sun X Ouyang X Li H Wei J . Increased IL-6 expression on THP-1 by IL-34 stimulation up-regulated rheumatoid arthritis Th17 cells. Clin Rheumatol. (2018) 37:127–37. doi: 10.1007/s10067-017-3746-y 28812210

[27] Hwang SJ Choi B Kang SS Chang JH Kim YG Chung YH . Interleukin-34 produced by human fibroblast-like synovial cells in rheumatoid arthritis supports osteoclastogenesis. Arthritis Res Ther. (2012) 14:R14. doi: 10.1186/ar3693 22264405 PMC3392804

[28] Arnett FC Edworthy SM Bloch DA McShane DJ Fries JF Cooper NS . The American Rheumatism Association 1987 revised criteria for the classification of rheumatoid arthritis. Arthritis Rheum. (1988) 31:315–24. doi: 10.1002/art.1780310302 3358796

[29] van Riel PL Renskers L . The Disease Activity Score (DAS) and the Disease Activity Score using 28 joint counts (DAS28) in the management of rheumatoid arthritis. Clin Exp Rheumatol. (2016) 34:S40–4. 27762189

[30] van der Heijde D . How to read radiographs according to the Sharp/van der Heijde method. J Rheumatol. (1999) 26:743–5. 10090194

[31] Blasioli DJ Matthews GL Kaplan DL . The degradation of chondrogenic pellets using cocultures of synovial fibroblasts and U937 cells. Biomaterials. (2014) 35:1185–91. doi: 10.1016/j.biomaterials.2013.10.050 24225084 PMC3877671

[32] Yang Z Hou N Cheng W Lu X Wang M Bai S . MiR-378 exaggerates angiogenesis and bone erosion in collagen-induced arthritis mice by regulating endoplasmic reticulum stress. Cell Death Dis. (2024) 15:910. doi: 10.1038/s41419-024-07193-5 39695085 PMC11655635

[33] Wang X Sun L An Z . CXCL7 enhances RANKL-induced osteoclastogenesis via the activation of ERK/NFATc1 signaling pathway in inflammatory arthritis. Arthritis Res Ther. (2025) 27:34. doi: 10.1186/s13075-025-03502-1 39955597 PMC11829549

[34] Li Y Fu Y Chen H . Blocking interleukin-33 alleviates the joint inflammation and inhibits the development of collagen-induced arthritis in mice. J Immunol Res. (2020) 2020:4297354. doi: 10.1155/2020/4297354 33490289 PMC7801941

[35] Qi X Meng J Li C Cheng W Fan A Huang J . Praelolide alleviates collagen-induced arthritis through increasing catalase activity and activating Nrf2 pathway. Phytomedicine. (2024) 135:156040. doi: 10.1016/j.phymed.2024.156040 39299092

[36] Yoshitomi H . Regulation of immune responses and chronic inflammation by fibroblast-like synoviocytes. Front Immunol. (2019) 10:1395. doi: 10.3389/fimmu.2019.01395 31275325 PMC6593115

[37] Xu H Jia Y Li J Huang X Jiang L Xiang T . Niloticin inhibits osteoclastogenesis by blocking RANKL-RANK interaction and suppressing the AKT, MAPK, and NF-κB signaling pathways. BioMed Pharmacother. (2022) 149:112902. doi: 10.1016/j.biopha.2022.112902 35364377

[38] Zheng H Liu Y Deng Y Li Y Liu S Yang Y . Recent advances of NFATc1 in rheumatoid arthritis-related bone destruction: mechanisms and potential therapeutic targets. Mol Med. (2024) 30:20. doi: 10.1186/s10020-024-00788-w 38310228 PMC10838448

[39] Zhang L Cui M Ding L Xia L Lu J Shen H . Interleukin-34 aggravates the severity of arthritis in collagen-induced arthritis mice by inducing interleukin-17 production. J Interferon Cytokine Res. (2018) 38:221–5. doi: 10.1089/jir.2017.0095 29664689

[40] Hussain A Tarahomi T Singh L Bollampally M Heydari-Kamjani M Kesselman MM . Cardiovascular risk associated with TNF alpha inhibitor use in patients with rheumatoid arthritis. Cureus. (2021) 13:e17938. doi: 10.7759/cureus.17938 34660128 PMC8513733

