# Supplementary material for: IL-34-mediated fibroblast-like synoviocyte-macrophage crosstalk drives bone erosion in rheumatoid arthritis through RANKL-dependent osteoclastogenesis
Source: Front Immunol. 2026 Jun 17;17:1822793. doi: 10.3389/fimmu.2026.1822793 (PMC13322645; doi:10.3389/fimmu.2026.1822793)
Supplement: Supplementary file 1 [file Table1.docx]

Table S1. Baseline demographic and clinical characteristics

| Characteristic | | RA (n = 42) | Control (n = 35) | P value |
| --- | --- | --- | --- | --- |
| Age (years, mean ± SD) | | 51.9 ± 10.3 | 47.8 ± 12.2 | 0.112 |
| Sex (male/female) | | 15/27 | 14/21 | 0.812 |
| Disease duration (years, median [IQR]) | | 8.0 (4.0–16.0) | N/A | – |
| DAS28‑ESR | | 4.393 ± 1.187 | N/A | – |
| DAS28‑CRP | | 4.020 ± 1.040 | N/A | – |
| Modified Sharp/van der Heijde score | | 19.79 ± 6.813 | N/A | – |
| RF positive, n (%) | | 31 (73.8%) | N/A | – |
| ACPA positive, n (%) | | 28 (66.7%) | N/A | – |
| Treatments | csDMARDs, n (%) | 35 (83.3%) | N/A | – |
|  | Biologics, n (%) | 6 (14.3%) | N/A | – |
|  | JAK inhibitors, n (%) | 2 (4.8%) | N/A | – |
|  | Glucocorticoids, n (%) | 18 (42.9%) | N/A | – |
| Comorbidities | Hypertension, n (%) | 12 (28.6%) | 8 (22.9%) | 0.591 |
|  | Diabetes, n (%) | 7 (16.7%) | 5 (14.3%) | 0.761 |

N/A = not applicable; – = no statistical test performed.
